# Suppression of Dendritic Cell-Derived IL-12 by Endogenous Glucocorticoids Is Protective in LPS-Induced Sepsis

**DOI:** 10.1371/journal.pbio.1002269

**Published:** 2015-10-06

**Authors:** Caiyi C. Li, Ivana Munitic, Paul R. Mittelstadt, Ehydel Castro, Jonathan D. Ashwell

**Affiliations:** Laboratory of Immune Cell Biology, National Cancer Institute, National Institutes of Health, Bethesda, Maryland, United States of America; National Jewish Medical and Research Center/Howard Hughes Medical Institute, UNITED STATES

## Abstract

Sepsis, an exaggerated systemic inflammatory response, remains a major medical challenge. Both hyperinflammation and immunosuppression are implicated as causes of morbidity and mortality. Dendritic cell (DC) loss has been observed in septic patients and in experimental sepsis models, but the role of DCs in sepsis, and the mechanisms and significance of DC loss, are poorly understood. Here, we report that mice with selective deletion of the glucocorticoid receptor (GR) in DCs (GR^CD11c-cre^) were highly susceptible to LPS-induced septic shock, evidenced by elevated inflammatory cytokine production, hypothermia, and mortality. Neutralizing anti-IL-12 antibodies prevented hypothermia and death, demonstrating that endogenous GC-mediated suppression of IL-12 is protective. In LPS-challenged GR^CD11c-cre^ mice, CD8^+^ DCs were identified as the major source of prolonged IL-12 production, which correlated with elevations of NK cell-derived IFN-γ. In addition, the loss of GR in CD11c^+^ cells rescued LPS-induced loss of CD8^+^ DCs but not other DC subsets. Unlike wild-type animals, exposure of GR^CD11c-cre^ mice to low-dose LPS did not induce CD8^+^ DC loss or tolerance to subsequent challenge with high dose, but neutralization of IL-12 restored the ability of low-dose LPS to tolerize. Therefore, endogenous glucocorticoids blunt LPS-induced inflammation and promote tolerance by suppressing DC IL-12 production.

## Abstract 2

Lipopolysaccharide (LPS) from bacteria causes the increased production of endogenous glucocorticoids, protecting mice from sepsis and contributing to LPS tolerance by suppressing production of interleukin-12 (IL-12) by dendritic cells and causing the death of the primary producers of IL-12. Read the Synopsis.

## Author Summary

Sepsis refers to life-threatening systemic inflammation, often caused by infection with bacteria that produce lipopolysaccharide (LPS). Glucocorticoids, immunosuppressive hormones produced by the adrenals, have been used to treat sepsis for over 50 y, but little is known about the role of endogenous (naturally occurring) glucocorticoids in systemic inflammation. Macrophages have been considered the primary source of inflammatory mediators (cytokines) and a target for glucocorticoid-mediated suppression. The possible role of another immune cell population, dendritic cells, has not been explored in detail. We created a mouse model in which the glucocorticoid receptor is selectively deleted in dendritic cells (DCs). We found that the elevation of glucocorticoids that accompanies sepsis protects mice from LPS-induced septic shock by suppressing DC production of IL-12, a cytokine that causes the secretion of other inflammatory mediators. In addition, LPS-induced glucocorticoids caused the death of a subset of DCs that are the primary producers of IL-12. Glucocorticoids were also found to be important for the phenomenon of "LPS tolerance", in which inoculation with low-dose LPS makes mice resistant to rechallenge with a high dose. This unexpected role of DC-produced IL-12 and its suppression by endogenous glucocorticoids may account, at least in part, for the known association of adrenal insufficiency and prolonged sepsis.

## Introduction

Sepsis is a complex clinical disorder arising from dysregulated systemic inflammatory responses. Severe sepsis and septic shock are a major cause of mortality among the critically ill. Early phase sepsis is characterized by exaggerated inflammatory cytokine production, also called cytokine storm, which can cause multiple organ dysfunction and death [1]. If the heightened inflammatory response is survived, compensatory mechanisms that attempt to control it eventually lead to profound immunosuppression, which can in turn result in lethal secondary infections [2]. The lack of understanding of the dynamic and heterogeneous mechanisms of this transition has hindered the development of effective immunoregulatory therapies for septic patients [3].

In sepsis caused by gram-negative bacteria, many of the life-threatening complications, such as hypercoagulation, hypothermia, and systemic inflammation are ascribed to lipopolysaccharide (LPS), also called endotoxin, a constituent of the bacterial cell wall [4]. Activation of innate immune cells by LPS via Toll-like receptor 4 (TLR4) initiates production of proinflammatory cytokines such as TNF-α, IL-1β, IL-6, IL-12, and IFN-γ. Although playing important roles in mounting effective immune responses to clear pathogens, overproduction of these cytokines leads to lethality mimicking the hyperinflammation of sepsis [5]. On the other hand, sublethal doses of LPS induce endotoxin tolerance, a temporary state of hyporesponsiveness of the innate immune system, which renders mice resistant to a subsequent lethal LPS challenge [6]. Originally recognized as a mechanism to limit the inflammatory response to persistent infections, endotoxin tolerance is now considered as a model for the study of postseptic immunosuppression, since a similar loss of LPS reactivity has been reported in circulating leukocytes of septic patients with elevated risk of succumbing to infection [7].

Glucocorticoids (GC) are steroid hormones that control a variety of essential metabolic, cardiovascular, and homeostatic functions [8]. GC are highly immunosuppressive and anti-inflammatory when given at pharmacologic levels [9,10]. The role of endogenous GC, when induced by stresses such as systemic inflammation and trauma, in controlling innate immune responses is not as well-characterized [11]. Observations supporting a role for endogenous GC in controlling sepsis are that adrenalectomized mice are more susceptible to LPS-induced septic shock [12], and adrenal insufficiency in humans is associated with prolonged sepsis [13,14]. On the other hand, adrenalectomized mice failed to develop tolerance, suggesting a GC-dependent mechanism of endotoxin tolerance [15]. Despite the importance of GC in the control of inflammatory disorders, it has been challenging to pinpoint the relevant cellular targets of endogenous GC in vivo, because the glucocorticoid receptor (GR) is ubiquitously expressed. There have been only a few studies using genetically altered GR to study the role of endogenous GC in sepsis. Mice in which the wild-type (WT) GR was replaced with a mutant with impaired dimerization, and therefore reduced gene transactivation ability (GR^dim^), were found to have higher mortality and elevated levels of TNF-α, IL-1β, and IL-6 compared to WT mice in two sepsis models, cecal ligation, and puncture and LPS [16]. A similar result was found when LPS was injected into conditional knockout animals in which the GR was deleted in macrophages or monocytes using lysM-cre, confirming a role of endogenous GC in the suppression of macrophages [17].

Primarily studied as antigen-presenting cells initiating adaptive immune responses, dendritic cells (DCs) are also essential orchestrators of innate immunity [18]. In contrast to macrophages, which have been the focus of sepsis and endotoxin tolerance research, the role of DCs in sepsis has not been extensively explored. In mice, two major subsets of splenic DCs can be defined, conventional DCs (cDCs) and plasmacytoid DCs (pDCs). cDCs are further divided into CD8^+^ and CD8^−^ populations, with the CD8^+^ DCs specializing in antigen cross presentation [19]. It has been shown that in LPS-challenged mice, CD8^+^ DCs are the major source of IL-12, one of the inflammatory cytokines elevated in LPS-induced sepsis [20]. IL-12 is a potent inducer of natural killer (NK) cell production of IFN-γ, high levels of which are associated with LPS-induced lethality [21,22]. However, unlike TNF-α and IL-1β, the contribution of IL-12 to LPS-induced lethality is controversial, particularly because injection of IL-12 alone does not induce shock [23]. In addition, DC loss is a hallmark of sepsis in both humans and animal models, seemingly arguing against a role of DCs as a major inflammatory mediator. The cause of DC loss remains elusive.

Using mice in which the GR has been selectively deleted in DCs (GR^CD11c-cre^ mice), we addressed the role of DCs and their regulation by endogenous GC in LPS-induced sepsis. The results indicate that endogenous GC mitigate LPS-induced lethality via suppression of DC-derived IL-12 and inflammatory cytokine production. Furthermore, the effects of GC on DCs are required for the establishment LPS-induced tolerance.

## Results

### GR^CD11c-cre^ Mice Are Highly Susceptible to LPS-Induced Lethality

**Fig 1:**
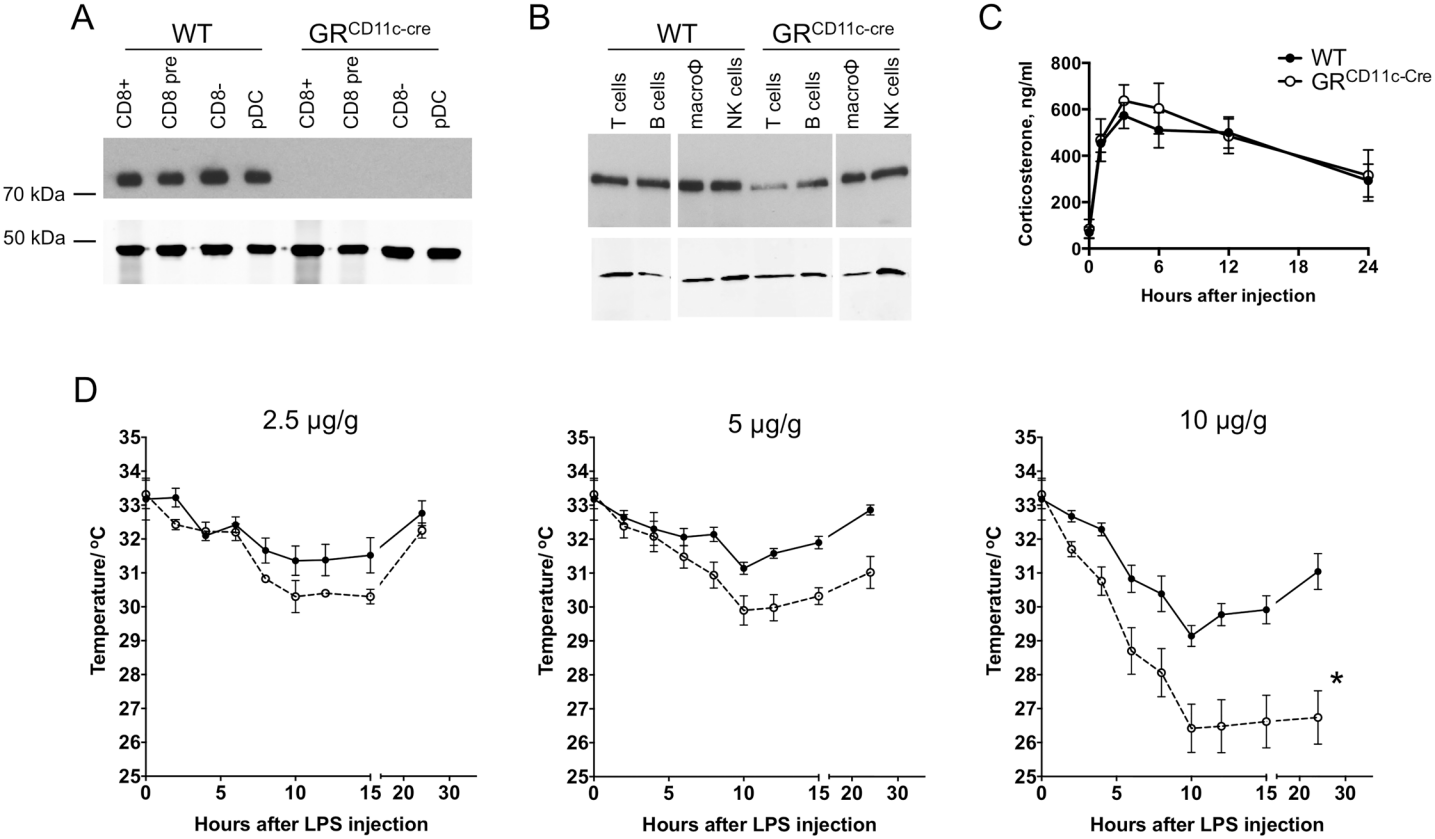
GR^CD11c-cre^ mice are more susceptible to LPS-induced septic shock than WT. (A) and (B) Immunoblot for GR in sorted DCs and other immune subsets from WT and GR^CD11c-cre^ mice. β-Actin was blotted as loading control. Some lanes were reordered for clarity, which is indicated by vertical white lines. (C) Plasma corticosterone concentrations after LPS challenge. Age- and sex-matched WT and GR^CD11c-cre^ mice were injected with LPS (3 μg/g body weight), and blood was drawn at indicated time points. Data shown are from four to eight animals per strain per time point pooled from multiple independent experiments. (D) GR^CD11c-cre^ mice (*n* = 4–6 per strain for each dose of LPS) were injected with the indicated amount of LPS, surface body temperature was recorded at the indicated times. * Four out of five GR^CD11c-cre^ mice died by 24 hr after LPS challenge. The data used to make this figure can be found in S1 Data.

GR^CD11c-cre^ mice were generated by crossing CD11c-cre transgenic mice with GR-floxed mice. Successful deletion of GR in sort-purified splenic DC subsets in GR^CD11c-cre^ mice was confirmed by immunoblotting (Fig 1A). In contrast, GR expression was normal in B cells, macrophages, and NK cells (Fig 1B). There was an ~50% reduction in T cell GR levels, presumably due to transient CD11c expression during thymocyte development [24]. WT and GR^CD11c-cre^ mice were injected with sublethal doses of LPS. We first confirmed that LPS challenge led to rapid elevation in circulating GC, the extent and kinetics of which were similar in mice of both genotypes (Fig 1C). Serum GC levels peaked at 3 hr and remained elevated for 24 hr. To compare the susceptibility to LPS-induced sepsis, mice were challenged with increasing doses of LPS, and the development of hypothermia as well as mortality was monitored (Fig 1D). Both WT and GR^CD11c-cre^ mice developed hypothermia in a dose-dependent manner, which was more pronounced in GR^CD11c-cre^ mice. When challenged with 10 μg/g body weight of LPS, the GR^CD11c-cre^ mice developed severe hypothermia, and four of five mice succumbed, whereas all WT mice (for which the lethal dose is ~25 μg/g) had milder hypothermia, and no death occurred. Thus, the GR^CD11c-cre^ mice are more susceptible to LPS-induced lethality than WT.

### Glucocorticoid-Mediated IL-12 Suppression Is Protective in LPS-Induced Sepsis

**Fig 2:**
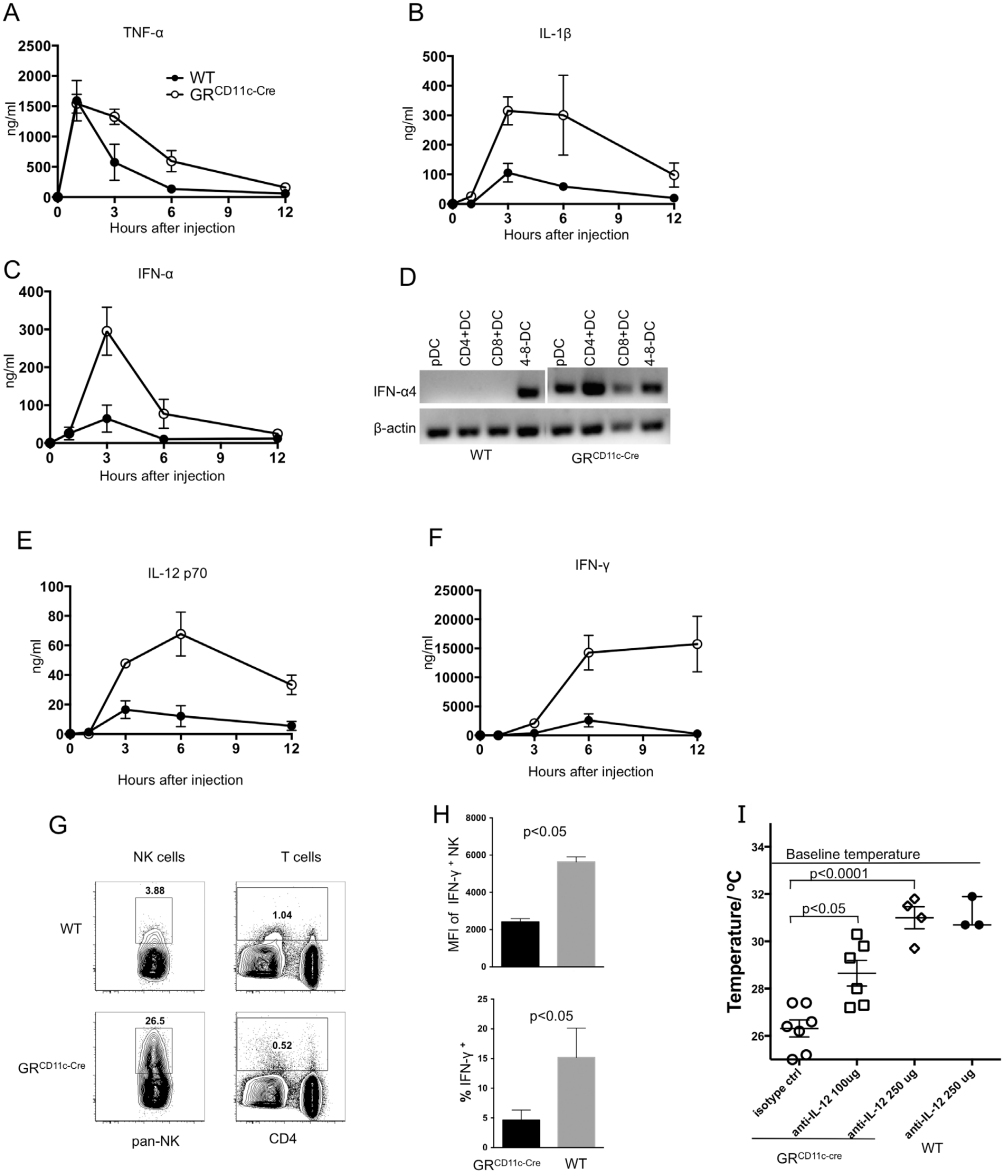
Elevated proinflammatory cytokines in GR^CD11c-cre^ mice. (A–C, E, and F) Kinetics of LPS-induced cytokine levels in WT and GR^CD11c-cre^ mice. The sera used in Fig 1C were also used to determine cytokine concentrations at each time point. Data shown are from four to eight animals per strain per time point pooled from multiple independent experiments. (D) Semiquantitative RT-PCR analysis of IFN-α4 transcription in DC subsets from WT and GR^CD11c-cre^ mice 1 hr after LPS challenge. β-actin was used as internal control. Results shown are one representative of two independent experiments with pooled splenocytes from two WT and two GR^CD11c-cre^ mice. (G) Splenocytes were prepared from mice 12 hr after LPS injection, and cultured in vitro for 4 hr with Brefeldin A. Intracellular IFN-γ was measured for gated NK and T cells by flow cytometry. One mouse from each genotype is shown out of two independent experiments, each using three mice per group. (H) Mean fluorescence intensity (MFI) and percentage of IFN-γ^+^ cells in WT and GR^CD11c-cre^ mice 12 hr post LPS challenge (*n* = 3). (I) GR^CD11c-cre^ or WT mice, each represented by an individual symbol, were injected with the indicated doses of neutralizing anti-IL-12 antibody 1 hr before challenge with a lethal dose of LPS (10 μg/g body weight). Surface body temperature at 30 hr is plotted. Results shown are pooled from two independent experiments, each with three to four mice per group. The data used to make this figure can be found in S1 Data.

To identify the mechanisms underlying the heightened susceptibility of GR^CD11c-cre^ mice to LPS, serum levels of cytokines that contribute to the severity of sepsis [23] were measured over time. Levels of all cytokines analyzed were increased in the sera of GR^CD11c-cre^ mice, and some had distinct kinetic differences compared to WT animals (Fig 2).

TNF-α and IL-1β are the most intensively studied inflammatory mediators of pathology in sepsis [23]. Serum TNF-α levels peaked within 1 hr of LPS challenge and decayed with similar kinetics in WT and GR^CD11c-cre^ mice (Fig 2A), which agrees with the notion that macrophages are the major source of early TNF-α production, and that macrophage GR levels are normal in GR^CD11c-cre^ mice. By 12 hr, serum levels of TNF-α neared baseline in both WT and GR^CD11c-cre^ mice. The levels of IL-1β, which peaked at 3 hr and decayed thereafter in WT animals, were substantially higher in GR^CD11c-cre^ mice at 3 hr and remained elevated up to 6 hr (Fig 2B), suggesting a more substantial contribution by DCs. Recently, Type I interferons (IFN) were shown to play an important role in LPS-induced sepsis [25]. Serum IFN-α levels peaked 3 hr after LPS challenge in both WT and GR^CD11c-cre^ mice, but the levels were 6-fold higher in the latter (Fig 2C). Semiquantitative RT-PCR analysis of sort-purified splenic DC subsets 1 hr after LPS injection confirmed that GR^CD11c-cre^ DCs produced higher levels of IFN-α (Fig 2D).

The levels of IL-12, which is predominately produced by CD8^+^ DCs [20], were substantially higher at 3 hr in GR^CD11c-cre^ mice (Fig 2E). In contrast to TNF-α and IFN-α, which were rapidly down-regulated in both WT and GR^CD11c-cre^ mice, serum IL-12 remained elevated in GR^CD11c-cre^ mice for up to 12 hr, a time at which it was undetectable in WT animals, suggesting that endogenous GC are required for the down-regulation of IL-12. Consistent with IL-12 being a potent inducer of IFN-γ production, serum IFN-γ levels in GR^CD11c-cre^ mice were strikingly higher than in WT and remained elevated for 12 hr, which correlated with more severe hypothermia (Fig 2F). Though NK cells are considered the major source of LPS-induced IFN-γ [26], bystander CD8^+^ T cells have been reported to produce IFN-γ early in bacterial infection [27]. Because GR levels were diminished in GR^CD11c-cre^ T cells, it is a formal possibility that GR^CD11c-cre^ T cells could also contribute to the elevated IFN-γ. However, 12 hr after LPS challenge, enhanced production of IFN-γ from NK cells, but not T cells, in GR^CD11c-cre^ mice was demonstrated by intracellular staining (Fig 2G and 2H). These results support the hypothesis that the loss of GC-mediated suppression of the DC–NK axis is responsible for the heightened LPS susceptibility of GR^CD11c-cre^ mice. To determine to what extent enhanced IL-12 contributes, neutralizing anti-IL12 antibodies were injected an hour before LPS challenge. Thirty hr after LPS injection, all of the isotype control-treated GR^CD11c-cre^ mice developed severe hypothermia and died. In contrast, anti-IL12 reduced hypothermia in GR^CD11c-cre^ mice in a dose-dependent manner, the small decrease in temperature at the higher dose being similar to that seen in WT mice (Fig 2I). These results demonstrate that endogenous LPS-induced GC are required to prevent lethal inflammation by suppressing IL-12 production.

### GC Suppress IL-12 Production by CD8^+^ DCs

**Fig 3:**
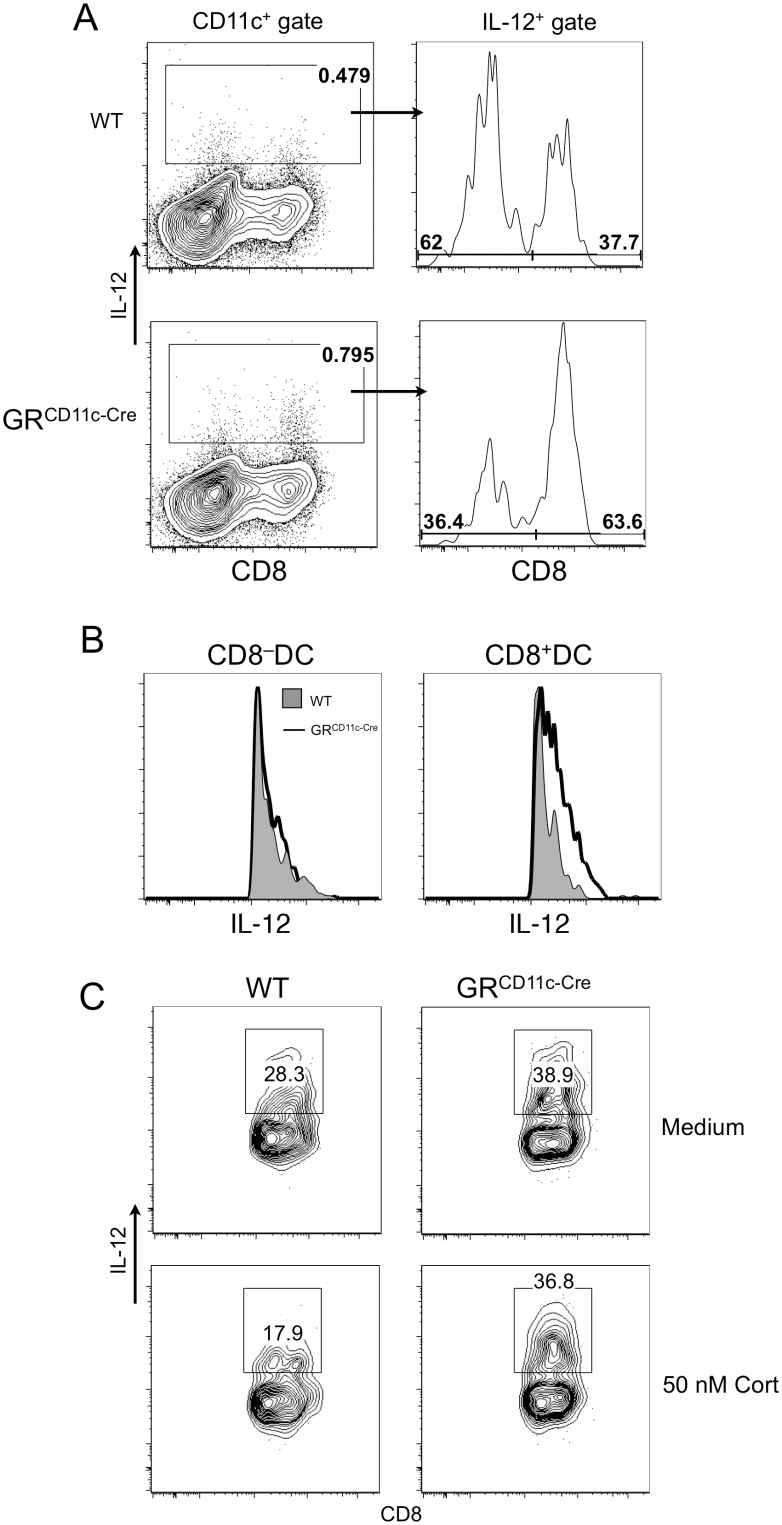
GC suppresses in vivo IL-12 production by CD8^+^ DCs. (A) Six hr after LPS challenge, WT and GR^CD11c-cre^ splenocytes were analyzed for intracellular IL-12 levels. The right panels are histograms of CD8 staining of CD11c^+^ IL-12^+^ cells. (B) Intracellular IL-12 levels in CD8^−^ and CD8^+^ DCs between WT (shaded) and GR^CD11c-cre^ (solid line). One representative pair of a group of three is shown. (C) GC suppression of IL-12 production by CD8^+^ DCs ex vivo. Splenocytes were prepared from mice 3 hr after LPS injection and cultured with Brefeldin A in the presence or absence of corticosterone for 4 hr. Intracellular IL-12 levels on gated CD8^+^ DCs from one experiment are shown. The data are representative of three independent pairs of mice.

CD8^+^ DCs are considered the major source of splenic IL-12 in mice exposed to LPS [20], but inflammatory DCs have also been shown to secrete IL-12 under certain inflammatory conditions [28]. To identify the DC subsets that are responsible for enhanced IL-12 production in the absence of GR, WT and GR^CD11c-cre^ mice were treated with LPS for 6 hr, and intracellular IL-12 levels were assessed for all CD11c^+^ cells. IL-12 was detected in only a small fraction WT CD11c^+^ cells, whereas GR^CD11c-cre^ had a 2-fold higher IL-12^+^ population (Fig 3A). Inflammatory Ly6C^+^ DCs were not major contributors (S1 Fig). Furthermore, a higher percentage of IL-12^+^ cells were CD8^+^ in the GR^CD11c-cre^ mice. Importantly, although there was no difference in IL-12 mean fluorescence intensity (MFI) in CD8^−^ cDCs between WT and GR^CD11c-cre^ mice, CD8^+^ cells in GR^CD11c-cre^ mice produced higher levels of IL-12 on a per cell basis (Fig 3B). In contrast, TNF-α production by DCs was not increased in GR^CD11c-cre^ cells (S2 Fig). These data suggest that among splenic cDCs that are induced to produce IL-12 by LPS, CD8^+^ DCs are uniquely sensitive to endogenous GC for IL-12 suppression, the absence of which led to prolonged elevation of serum IL-12 in GR^CD11c-cre^ mice. To directly test this hypothesis, splenocytes were isolated from mice treated with LPS for 3 hr and cultured with or without corticosterone for an additional 3 hr, to mimic the effect of endogenous GC. As shown in Fig 3C, IL-12 production was inhibited in WT but not GR^CD11c-cre^ CD8^+^ DCs. Therefore, one function of endogenous GC induced by LPS is to directly suppress IL-12 production by CD8^+^ DCs.

### LPS-Induced Loss of CD8^+^ DCs Is GC-Mediated

**Fig 4:**
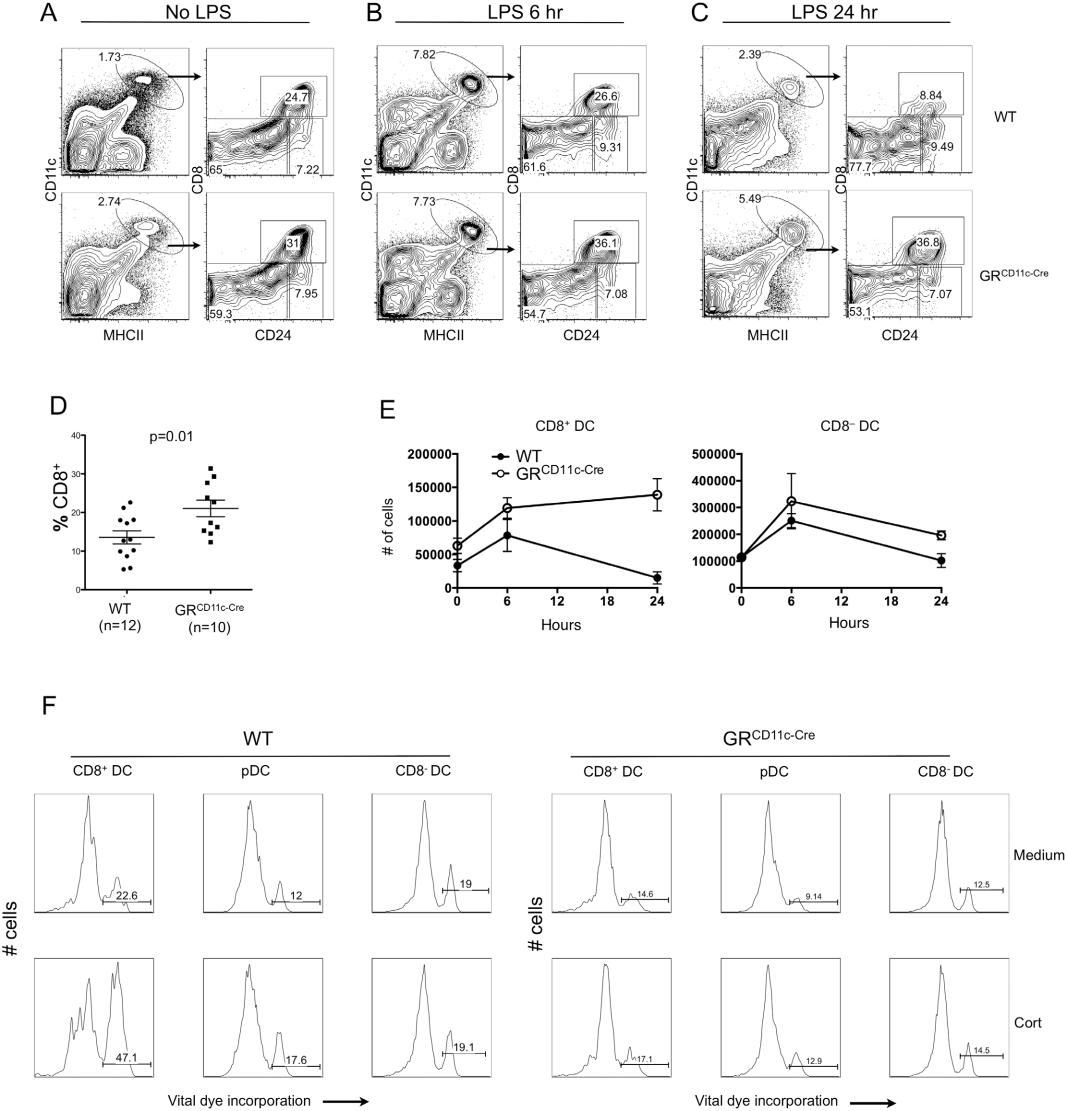
LPS-induced loss of CD8^+^ DCs is GR-dependent. (A–C) Splenic DCs from WT and GR^CD11c-cre^ B10.A mice were analyzed by flow cytometry at indicated times after injection with LPS (3 μg/g body weight). Only B220^−^TCRβ^−^ cells are shown. Results shown are from one representative experiment of three independent ones, each with three to five mice per group (D) Percentage of CD8^+^ DCs in WT and GR^CD11c-cre^ mice at time 0 (steady state). Results shown are from three pooled independent experiments, with 3–4 mice per group. (E) Total number of CD8^+^ and CD8^−^ DCs at time 0, 6 hr, and 24 hr post LPS injection. Results shown are from one representative experiment of three independent ones, each with three to five mice per group. (F) Sensitivity of DC subsets to GC-induced death in vitro. Total splenocytes from WT and GR^CD11c-cre^ mice were incubated with 100 nM corticosterone in vitro for 6 hr, and the percentage of dead cells in indicated populations are shown. The data shown are representative of four pairs of mice in two independent experiments. The data used to make this figure can be found in S1 Data.

LPS is known to induce DC loss in vivo [29], but the mechanism is not well understood. In steady-state GR^CD11c-cre^ mice, although DC activation as measured by surface expression of CD80 and CD86 was similar to WT (S3 Fig), there was a statistically significant increase in the fraction of CD8^+^ DCs (Fig 4A and 4D). A possible explanation for this is that physiologic fluctuations of endogenous GC might affect the lifespan of CD8^+^ DCs. Indeed, endogenous GC-dependent CD8^+^ DC loss was reported in mice subjected to physical restraint [30], prompting us to ask if LPS-induced DC loss is caused by endogenous GC. Splenic DC populations were analyzed at different time points following LPS challenge. Six hr post challenge, total DC and CD8^+^ DC numbers were increased in both WT and GR^CD11c-cre^ mice (Fig 4B and 4E), an effect that corresponds to LPS-induced DC maturation [29]. Twenty-four hr after LPS injection, a substantial loss of both CD8^−^ and CD8^+^ DCs was observed in WT mice. Strikingly, in GR^CD11c-cre^ mice, although the loss of CD8^−^ DCs resembled that in WT animals, the loss of CD8^+^ DCs was completely prevented (Fig 4C and 4E). These results demonstrate that endogenous GC are responsible for LPS-induced CD8^+^ DC loss in vivo. To test if CD8^+^ DCs are directly sensitive to GC-mediated killing, steady state splenocytes were incubated in the presence of corticosterone and cell death of DC subsets was analyzed by flow cytometry (Fig 4F). After 6 hr of exposure to corticosterone, WT CD8^−^ DC viability was unaffected, and that of pDCs was only modestly reduced. In contrast, CD8^+^ DC viability was markedly reduced. As expected, GR^CD11c-cre^ CD8^+^ DCs were completely refractory to GC-induced death. These results suggest DC subset-specific sensitivity to endogenous GC, with CD8^+^ DCs being the most sensitive, and demonstrate that LPS-induced loss of CD8^+^ DCs is GR-dependent.

### Loss of Endotoxin Tolerance in GR^CD11c-cre^ Mice

**Fig 5:**
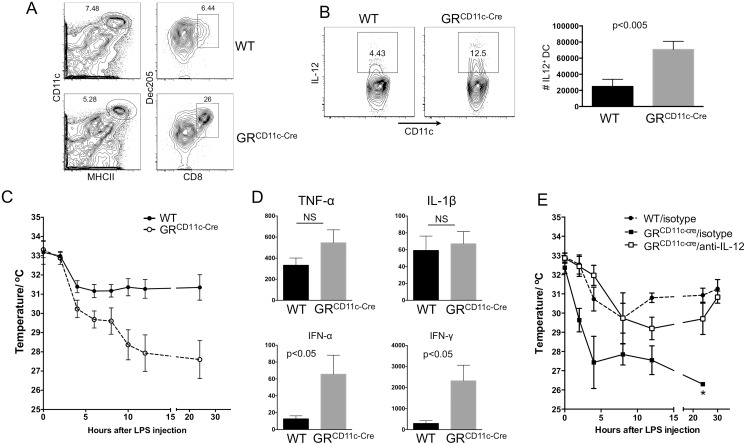
Loss of endotoxin tolerance in GR^CD11c-cre^ mice. (A) Tolerizing dose of LPS-induced loss of CD8^+^ DCs in WT but not GR^CD11c-cre^ mice. Mice were treated with 0.5 μg/g body weight of LPS for 24 hr and rechallenged with 3 μg/g body weight of LPS for 3 hr before splenic DCs were analyzed by flow cytometry. The experiment was repeated three times, with three mice in each group per experiment. (B) High-dose LPS induced IL-12 production by DCs in GR^CD11c-cre^ mice despite prior exposure to low-dose LPS. Ten to twelve wk female GR^CD11c-cre^ mice (*n* = 4) and control mice (*n* = 5) were treated with 0.5 μg/g body weight of LPS for 24 hr and rechallenged with 10 μg/g body weight of LPS for 6 hr. Percentage of IL-12^+^ DCs in the spleen of a representative mouse is shown (left panel). Average numbers of total IL-12^+^ DCs were calculated based on the total number of splenocytes in each mouse (right panel). (C) Body temperature of mice rechallenged with 10 μg/g body weight of LPS 24 hr after tolerization. Results were pooled from two independent experiments, *n* = 8 for WT and *n* = 5 for GR^CD11c-cre^. (D) Serum levels of TNF-α, IL-1β, IFN-α, and IFN-γ from mice in (B) were measured 6 hr after rechallenge. (E) Body temperature of mice rechallenged with 10 μg/g body weight LPS 24 hr after tolerization and 1 hr after injection with 250 μg anti-IL-12 or isotype control. Results were pooled from two independent experiments with one to two mice per condition, respectively, *n* = 3 per condition. The asterisk indicates the time by which all GR^CD11c-cre^ mice had died. All WT and the GR^CD11c-cre^ animals treated with anti-IL-12 survived. The data used to make this figure can be found in S1 Data.

Endotoxin tolerance in vivo is associated with a reduction in LPS-induced IL-12 and IFN-γ production [31]. Loss of IL-12 induction in mice has been correlated with DC loss, although the cause of DC loss during low-dose LPS challenge was not determined [32]. Our findings imply that endogenous GC induced by a tolerizing dose of LPS might be the cause of CD8^+^ DC loss, which then account for the lack of IL-12 induction after the rechallenge. In fact, LPS given at a dose that induces tolerance of IL-12 induction, 0.5 μg/g body weight [32], led to CD8^+^ DC loss in WT but not GR^CD11c-cre^ mice, similar to what was observed with higher dose LPS (compare Fig 5A to 4C). To test if the "LPS-tolerized" CD8^+^ DCs in GR^CD11c-cre^ mice can produce IL-12 upon rechallenge, mice were given high-dose LPS 24 hr after treatment with low-dose LPS. Six hr later, IL-12^+^ DCs were readily detectable in GR^CD11c-cre^ but not WT mice (Fig 5B). Therefore, the absence of the GR in DCs prevented CD8^+^ DC loss and rescued IL-12 production upon LPS rechallenge. This cellular observation correlated with the clinical response, in which low-dose LPS reduced hypothermia in WT mice after high-dose LPS rechallenge but failed to do so in GR^CD11c-cre^ mice (Fig 5C). The lack of tolerance in GR^CD11c-cre^ mice was correlated with statistically significantly higher levels of serum IFN-γ and IFN-α, though serum IL-12 was under the detection limit of our assay (Fig 5D). These mice also had modestly elevated serum levels of TNF-α, but IL-1β levels were comparable between WT and GR^CD11c-cre^ mice, suggesting that IL-12 and IFN-γ are largely responsible for the increased morbidity in the GR^CD11c-cre^ mice. The role of elevated IL-12 as the cause for lack of tolerance was confirmed with neutralizing anti-IL-12 antibodies (Fig 5E). After receiving tolerizing doses of LPS, all isotype control-treated GR^CD11c-cre^ mice succumbed to high-dose rechallenge, whereas the mice that received anti-IL-12 survived, with temperature curves similar to that of WT animals. Therefore, endogenous GC-mediated suppression of DC-derived IL-12 is also an essential mechanism of endotoxin tolerance in vivo.

## Discussion

Despite the primary experimental focus on macrophages in mechanistic studies of sepsis, there is scattered evidence that the DC/NK axis contributes as well. Early studies showed that IL-12 was required for IFN-γ production and lethality in LPS-induced septic shock [22]. More recently, mice with a gain-of-function Lyn mutation were shown to be highly susceptible to LPS [33]. Similar to GR^CD11c-cre^ mice, these animals manifested elevated levels of TNF-α, IL-6, IL-1β, IL-12, IFN-γ, and IFN-α, and DCs and NK cells were identified as the major sources of IL-12 and IFN-γ, respectively. Although no elevation in IL-12 and IFN-γ was observed in LPS-treated GR^dim^ mice [16], recent evidence shows that GR^dim^ is still able to repress proinflammatory gene transcription through a mechanism that is independent of the dimerization domain [reviewed by 34], leaving open the question of whether IL-12 production by DCs is regulated by endogenous GC during sepsis. We have answered this question by targeted deletion of GR in DCs and demonstrating that LPS-induced GCs also control the activation of the DC/NK cell axis, the failure of which leads to lethal inflammation. These results complement the study using the GR^LysM-cre^ mice and imply that in patients with compromised GC responses, such as in adrenal insufficiency, DCs can be a major source of inflammatory cytokines, and are in agreement with the association of sepsis with adrenal insufficiency and the finding that low-dose corticosteroids reduce mortality from septic shock in a subset of patients with adrenal insufficiency [35].

DC loss is considered a hallmark of sepsis-induced immune dysfunction in humans [36,37]. However, the cause of this loss is poorly understood. One possibility, that TLR signaling directly kills DCs, is controversial. Whereas some consider TLR4 signaling to be prosurvival for DCs [38], others have reported proapoptotic roles of TLR2 and TLR4 in polymicrobial sepsis. For example, in TLR2 and TLR4 double knockout mice, LPS-induced loss of CD8^−^ DCs was prevented. Nevertheless, CD8^+^ DCs were still lost in these mice, indicating that there is a TLR-independent mechanism as well [39]. Other mechanisms that have been proposed include death due to perforin and Fas [40], type I IFN [41], and LPS–CD14 interactions [42]. The GR^CD11c-cre^ mice have allowed us to establish that the loss of CD8^+^ DCs, but not CD8^−^ DCs, is due to endogenous GC that are up-regulated during sepsis. It is tempting to speculate that the loss of DCs in humans, just as in mice, is due to elevated endogenous GC. Although the identity of the human counterpart of mouse CD8^+^ DCs is in dispute [43–45], the suppression of DC-produced IL-12 by GC may be an important component of the innate immune response and contributes to clinical outcome.

The molecular mechanisms that confer the unique sensitivity of CD8^+^ DCs to GC are unknown. A recent study suggests that DC activation leads to a change in the expression of GR isoforms, which renders mature DCs more susceptible to GC than immature DCs [46]. On the other hand, GC induction by physical restraint induced CD8^+^ DC loss without evidence of DC activation [30], arguing that CD8^+^ DCs are intrinsically sensitive to GC. The data presented here support the latter interpretation. Genome-wide microarray analyses have identified several apoptotic regulators, including Bim and GILZ, in the regulation of GC-induced apoptosis [reviewed in 47]. A recent study comparing subset-specific transcriptomes of murine splenic CD8^+^ and CD11b^+^ DC subsets found that at steady state, CD8^+^ DCs had 3- to 26-fold higher levels of transcripts encoding the proapoptotic genes *Bad*, *Bim*, and *BCL2l14* (*BclG*) [48]. Moreover, 24 hr after LPS challenge mRNA encoding the anti-apoptotic *Bcl-xL* was 20-fold lower in CD8^+^ compared to CD11b^+^ DCs. We think it is likely that such differences contribute to the enhanced sensitivity of CD8^+^ DCs to GC.

The cellular and molecular mechanisms responsible for endotoxin tolerance are multifactorial and likely involve negative feedback at multiple levels in multiple cell types [7]. Although demonstrable both in vitro and in vivo, mechanistic studies have been reductive, with a primary focus on isolated monocytes and macrophages in vitro [6] in the absence of GC, despite the early observation of a GC-dependent mechanism of endotoxin tolerance in vivo [15]. Early evidence that macrophages were the target for clinical tolerance comes from studies in which unmanipulated macrophages were transferred into LPS-tolerized mice [49]. These animals developed sepsis when rechallenged with LPS (i.e., nontolerant macrophages broke whole animal tolerance). Here we provide insights into the relevance and mechanism of DC tolerance by showing that GC-mediated repression of DC proinflammatory cytokine production is an important aspect of clinical tolerance to endotoxin in vivo. The failure of endogenous GC to eliminate CD8^+^ DCs in GR^CD11c-cre^ mice given low-dose LPS correlates with the lack of tolerance, both in terms of IL-12 and IFN-γ production and the clinical response. It is interesting that low dose LPS caused little morbidity, even in GR^CD11c-cre^ animals, but still induced CD8^+^ DC loss, suggesting that the amounts of endotoxin needed to elevate GC to a biologically active level are lower than those needed to trigger a cytokine response. Formal proof that the loss of CD8^+^ DCs is important in LPS tolerance would require adoptive transfer of GR^CD11c-cre^ CD8^+^ DCs into WT mice. Experiments to this end have failed due to poor engraftment of the transferred cells, which has been observed by others [50]. Nonetheless, the fact that IL-12 plays a key role in both LPS sepsis and tolerance, and that we found CD8^+^ DCs were the major producers of IL-12 strongly suggests that this subset is a critical target for GC in mice.

The unique roles of DCs as both inflammatory response mediators and initiators of adaptive immune response provides a cellular basis for the proposal of endogenous GC as a “double-edged sword” as sepsis progresses. The initial GC-mediated suppression of cytokine production is necessary to prevent a cytokine storm and subsequent organ failure. Whereas GC-mediated loss of DCs might be expected to terminate an overly aggressive innate immune response, it may also contribute to postseptic immunosuppression, especially given the essential role for DCs in antigen presentation [19]. A critical balance between these activities of GC, in addition to other pro- and anti-inflammatory mediators, may determine the clinical outcome of sepsis.

## Materials and Methods

### Mice

C57BL/6 (B6), B10.A, CD11c-cre transgenic (B6^CD11c-cre^), and RAG2 knockout mice were obtained from Jackson Laboratory. Mice harboring a GR-floxed allele have been previously described [51]. B6 GR^fl/fl^ mice were backcrossed onto B6 for nine generations and then crossed with B6^CD11c-cre^ to generate GR^CD11c-cre^ mice. Age- and sex-matched littermate control mice were used in all experiments. All animals were maintained at an American Association for the Accreditation of Laboratory Animal Care-accredited and specific pathogen-free facility at the National Cancer Institute. This study was performed in strict accordance with the recommendations in the Guide for the Care and Use of Laboratory Animals of the National Institutes of Health. The protocol was approved by the NCI Animal Care and Use Committee (Protocol Number: LICB-039).

### LPS Treatment In Vivo

LPS from *Escherichia coli* (serotype 055:B5, Sigma #L2880) was solubilized in PBS and injected intraperitoneally (i.p.) into female mice at the doses specified in each experiment. Control mice were injected with PBS alone. Surface body temperature was measured with an infrared thermometer (VWR, #36934–182). For in vivo IL-12 neutralization, the indicated amounts of anti-IL-12 antibody or isotype control (Biolegend) was injected i.p. 1 hr before LPS injection. Mice that became moribund during the course of the study were euthanized and considered to be LPS-induced mortalities.

### Serum Cytokine and Corticosterone Measurement

When needed, blood samples were collected at one time point post LPS injection and again at the end point for each mouse. Serum GC levels were measured with Corticosterone Chemiluminescent Immunoassay kit (Arbor Assay). Serum levels of TNF-α, IFN-α, IL-1β, IL-10, IL-12, and IFN-γ were measured with FlowCytomix Simplex kits according to manufacturer’s protocol (eBiosciences/Affymetrix).

### Cell Preparation and Culture Condition

Single cell suspensions were prepared from spleens of individual naïve or LPS-injected mice by passage through nylon mesh strainers. Red blood cells were lysed before total splenocytes were enumerated. When indicated, cells were cultured with RPMI 1640, supplemented with 10% fetal calf serum, 5 mM glutamine, 5 μMβ-mercaptoethanol, and 100 μg/ml gentamicin. Corticosterone was purchased from Sigma and reconstituted in ethanol to 1 mM stock.

### Flow Cytometry and Intracellular Staining

Five million splenocytes from each mouse were stained in 100 μl of FACS buffer (PBS with 2% FBS), with Fixable Live/Dead Stain (Invitrogen/Life Technologies) and an appropriate combination of fluorescent antibodies specific for CD11c, MHC class II, CD4, CD8, CD86, CD80, TCRβ, B220, CD11b, NK1.1, pan-NK (anti-CD49b), and 2.4G2 (FcR block) (BD Pharmingen), for CD24, Dec205, PDCA, CD64, and Ly6C (Biolegend). For intracellular staining, 5 x 10^6^ splenocytes were cultured in six-well plates with 5 μg/ml of Brefeldin A (Sigma) for 4 hr before being harvested for cell surface staining, after which cells were fixed and permeabilized with Cytofix/Cytoperm (BD Pharmingen) and stained with PE-anti IL-12 p40/p70 (BD Pharmingen, #562038) and APC-anti-IFN-γ (BD Pharmingen, #554413). In some experiments, the cells were stained directly after isolation and without culture. Flow cytometry was performed with a BD LSRFortessa cytometer using BD FACSDiva software (BD Biosciences). Flow cytometry data analysis was performed with FlowJo software (Tree Star).

### Cell Sorting and Immunoblotting

All cells are sorted from single-cell suspension of spleens. Gating strategy for DC subsets was as follows: pDCs (B220^+^PDCA^+^CD11c^int^), CD8^+^ DCs (MHCII^hi^CD11c^hi^ CD8^+^Dec205^+^), CD8^+^ DC precursors (MHCII^hi^CD11c^hi^ CD8^−^CD24^+^) [50], and CD8^−^ DCs (MHCII^hi^ CD11c^hi^ CD8^−^CD24^−^). For other immune subsets: T cells (TCRβ^+^B220^−^), B cells (B220^+^ TCRβ^−^), macrophages (CD11b^+^ F4/80^+^), and NK cells (NK1.1^+^Pan NK^+^). Subsets of DCs and other immune cells were sort-purified on a FACSAria III (BD), and purity was confirmed by postsort analysis. Cells were normalized by number and lysed in sample buffer (50 mM Tris pH 6.8, 10% glycerol, 2% SDS, 2% β-mercaptoethanol, and 0.04% bromophenol blue), resolved with NuPAGE SDS-PAGE gels, transferred to nitrocellulose membranes, and immunoblotted with the antibody to the N-terminus of GR, M20 (Santa Cruz). Anti-β-Actin antibody was from Sigma.

### Semiquantitative RT-PCR

Total RNA was isolated from sorted DC subsets with RNeasy micro kit (Qiagen), and 25 ng of total RNA from each sample was used to amplify gene-specific mRNA with One-step RT-PCR kit (Qiagen). The following primer pairs were used: IFN-α4: 5’-GCAGAAGTCTGGAGAGCCCTC-3’ (forward), and 5’-tgagatgcagtgttctggtcc-3’(reverse); β-actin: 5’- CTAAGGCCAACCGTGAAAAG-3’(forward), and 5’-ACCAGAGGCATACAGGGACA-3’(reverse). PCR products were visualized on a 2% agarose gel.

### Statistical Analysis

Statistical analysis was done using Student's *t* test with GraphPad Prism software. Error bars represent SEM.

## Supporting Information

S1 Data Excel data file of numerical values that were used to generate graphs. (XLSX)

S1 Fig No induction of IL-12 in inflammatory Ly6C^hi^ DC in WT or GR^CD11c-cre^ mice. WT and GR^CD11c-cre^ mice were either injected or not with LPS (3 μg/g mouse weight), and at the indicated times splenocytes were stained for Ly6C and intracellular IL-12. The numbers in the gated areas represent the percent of cells. The experiment was repeated two times with three mice in each group.
(TIF)

S2 Fig Similar induction of TNF-α in DC from WT and GR^CD11c-cre^ mice. WT and GR^CD11c-cre^ mice were either injected or not with LPS (3 μg/g mouse weight), and at the indicated times splenocytes were stained for cell surface CD11c and intracellular TNF-α. The numbers in the gated areas represent the percent of cells. The experiment was repeated two times with three mice in each group.
(TIF)

S3 Fig Similar activation status of freshly harvested DC in WT and GR^CD11c-cre^ mice. Splenocytes were stained and gated on DC, and the activation markers CD86 and DEC205 on wild type (shaded) and GR^CD11c-cre^ (solid line) are shown. The data is representative of three independent experiments with three to five mice in each group.
(TIF)

## Abbreviations

cDC: conventional dendritic cell
DC: dendritic cell
GC: glucocorticoids
GR: glucocorticoid receptor
IFN: interferons
LPS: lipopolysaccharide
MFI: mean fluorescence intensity
NK: natural killer
pDC: plasmacytoid dendritic cell
TLR4: Toll-like receptor 4
WT: wild-type
