# Supplementary figures and images for: Suppression of Dendritic Cell-Derived IL-12 by Endogenous Glucocorticoids Is Protective in LPS-Induced Sepsis
Source: PLoS Biol. 2015 Oct 6;13(10):e1002269. doi: 10.1371/journal.pbio.1002269 (PMC4595142; doi:10.1371/journal.pbio.1002269)

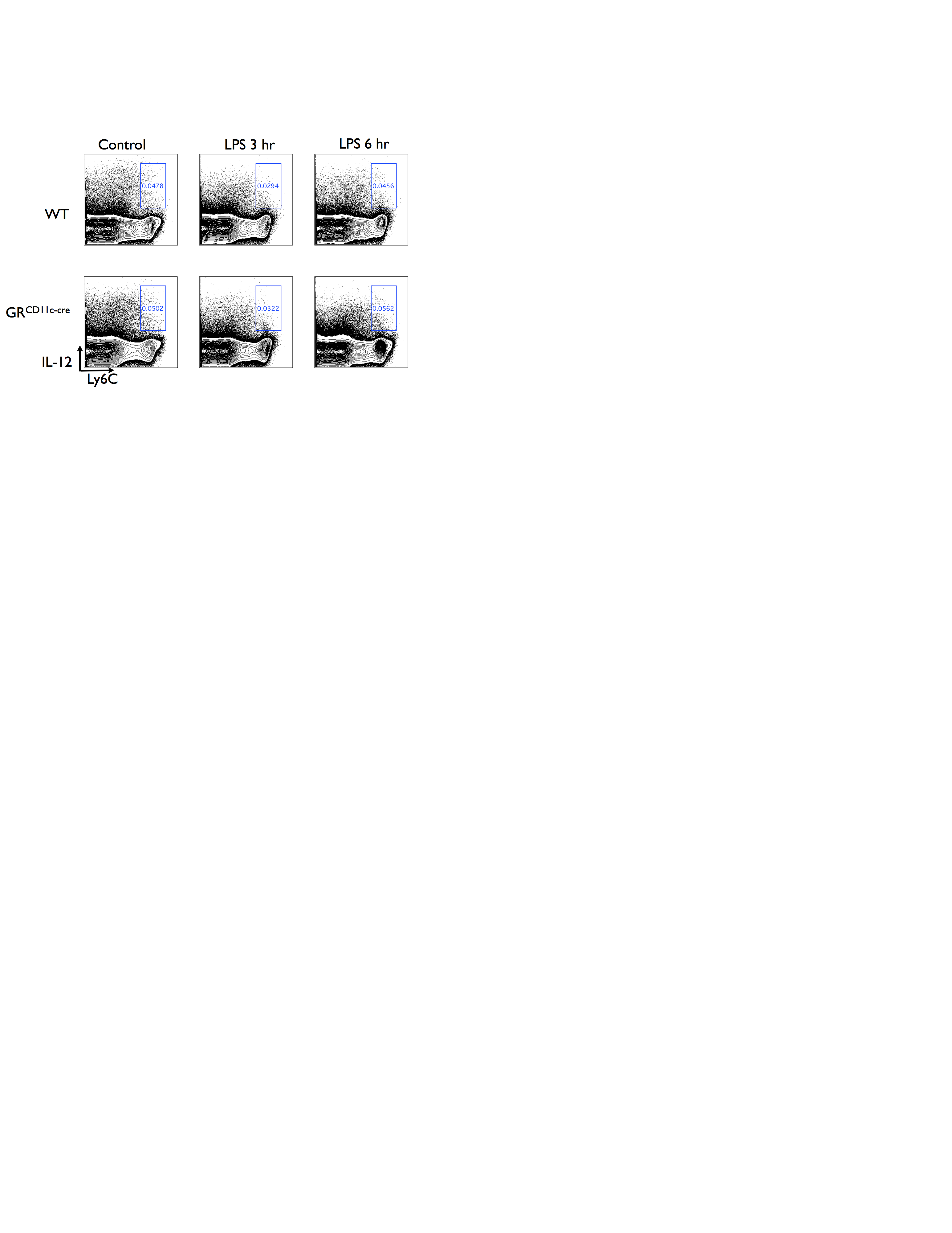

Supplement: S1 Fig — WT and GRCD11c-cre mice were either injected or not with LPS (3 μg/g mouse weight), and at the indicated times splenocytes were stained for Ly6C and intracellular IL-12. The numbers in the gated areas represent the percent of cells. The experiment was repeated two times with three mice in each group. (TIF) [file pbio.1002269.s002.tif]

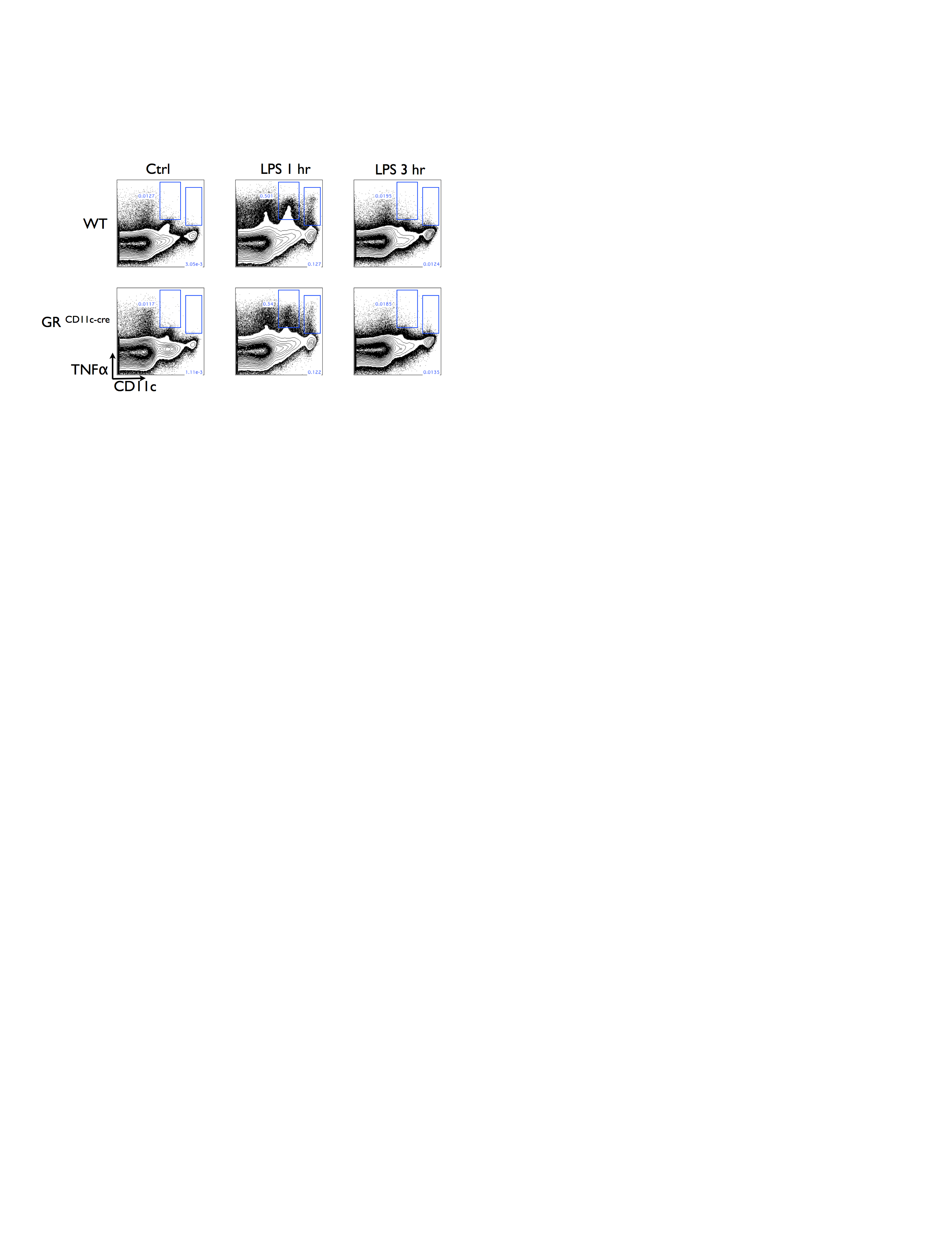

Supplement: S2 Fig — WT and GRCD11c-cre mice were either injected or not with LPS (3 μg/g mouse weight), and at the indicated times splenocytes were stained for cell surface CD11c and intracellular TNF-α. The numbers in the gated areas represent the percent of cells. The experiment was repeated two times with three mice in each group. (TIF) [file pbio.1002269.s003.tif]

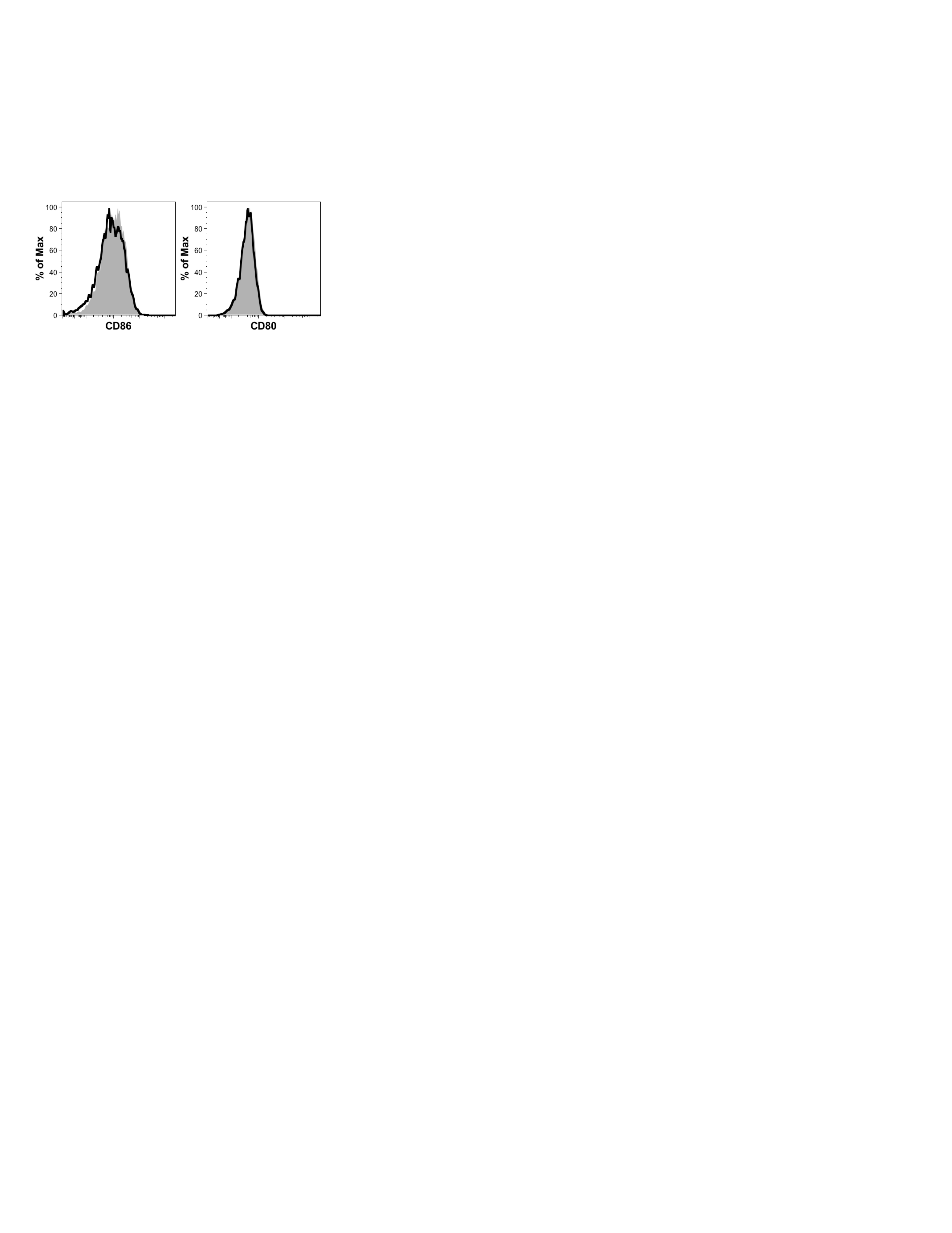

Supplement: S3 Fig — Splenocytes were stained and gated on DC, and the activation markers CD86 and DEC205 on wild type (shaded) and GRCD11c-cre (solid line) are shown. The data is representative of three independent experiments with three to five mice in each group. (TIF) [file pbio.1002269.s004.tif]
